# Hospital Reports

**Published:** 1838

**Authors:** 


### WESTERN JOURNAL

OF THE

### MEDICAL AND PHYSICAL SCIENCES.

**For January, February, and March, 1838.**

### ESSAYS AND CASES.

### Art. I.—Clinical Reports.

*Notes of Cases, with occasional remarks.* Arranged by the Editorial Committee.

### Encephaloid Eye-Ball.

Mrs. M. A. J., of Wisconsin Territory, entered the *Cincinnati Eye Infirmary*, on the 22d of October, 1837. Her age was about 42. She related that several years before, she had received a blow on her right eye, the lids being closed at the time, which was followed by severe pain, and permanent blindness, though no great degree of inflammation supervened. The pain ceased, and she enjoyed tolerable health; but in the month of January, 1837, she was again seized with excruciating pain in the globe of the eye, and a small discolored spot appeared on the sclerotica, between the cornea and the inner canthus. The pain recurred in paroxysms, and very soon a tumour began to appear, where the spot had shewn itself. Its growth was rapid, but irregular. For awhile it would lie dormant, but when at night acute pain occurred, it would always be found sensibly enlarged the next morning. Its form was nearly cylindrical, and in a few months it projected far beyond the eyelids, and assumed a livid aspect. In this condition, about midsummer, a physician applied some kind of escharotic to its extremity, which produced a foul ulcer, followed by repeated hæmorrhages. In October, when she applied to Prof. Drake, and was admitted into the Infirmary, it projected at least an inch and a half beyond the eye lids, with a diameter of nearly the same extent; but could be moved by the muscles of the eye ball, and did not appear to be attached to the socket. The lids, especially the upper, were tumid, and loaded with dark-colored blood. The eye ball was turned downwards and outwards, and completely hidden by the lower lid, which on being depressed near the outer angle disclosed the cornea, iris and pupil. Her general health was rather infirm; and she had occasional chills, followed by fever and great aggravation of pain in tumour, orbit and head.

All the essential symptoms of Fungus Hæmatodes being present, the question of the utility of the extirpation, was the only one which could be discussed. A consultation of the professors being held, it was communicated to the patient, that without an operation she would certainly die, and that the disease would probably be reproduced, after all that could be removed had been taken away. It was, also, explained to her that the operation *might* be followed by fatal inflammation of the brain. Under these discouraging circumstances, however, she chose to have it performed, but for reasons not connected with the disease it was deferred for three weeks.

When about to proceed to the operation, Prof. D. proposed to remove the upper eyelid, as being unsound, but was persuaded to limit the extirpation to the removal of the contents of the socket. This was done with a scalpel, ground to a circular, instead of a pointed extremity, so as to act on the principle of a spade, by which the danger would be averted, of piercing the roof of the socket, should it be carious or greatly attenuated, which, however, proved not to be the case; for all the parts connected with the socket, adispose and muscular, were healthy, except the optic nerve, which although cut as low down as possible, appeared to be unsound. The tumour was dissected by Prof. Gross, who made upon it the following report:

The entire mass, after it was divested of the muscles and celluloadipous tissue of the orbit, which were quite healthy, was two inches and three quarters in length, and five and a quarter in circumference, its weight being a little upwards of two ounces. The eye itself was of the ordinary form and volume, but was considerably thrown out of its position by the morbid growth, which was of an irregularly oval shape, and sprung from the inner side of the sclerotic coat, near its junction with the cornea. This connexion, however, was rather apparent than real; for upon tracing the heterologous mass, it was evident that it originated in the retina, which had itself almost totally disappeared. The anterior surface was closely invested by the conjunctiva, which had a rough, fleecy aspect, and about its centre was an incrusted ulcer three-fourths of an inch in diameter, around which the parts were somewhat knobby, and of a bluish livid color. On cutting through this portion of the tumor, it was found to consist essentially of vessels, some of which had been opened by the ulcerative process, and formed the source of the frequent hemorrhages with which the patient had been latterly affected. Posteriorly the mass was of a much lighter color, as well as more soft, and presented that peculiar tuberoid arrangement so characteristic of encephaloid.

The cornea, although still transparent, was considerably diminished in size, and adhered firmly to the iris. The sclerotic part was of the natural thickness, extensively attached to the choroid, and of a yellowish buff color. The choroid itself had a speckled, brownish appearance, and there were some points at which it was completely disorganized. At one part, nearly opposite the diseased mass, a black layer of blood was found beneath it. The retina, as was before stated, was almost entirely gone; and, in place of the vitreous humour, there was a dense, solid, white mass, evidently the result of an effusion of fibrin. The anterior chamber of the eye was obliterated, and the iris was transformed, in great measure, into a substance resembling fibro-cartilage. The optic nerve, near its entrance into the sclerotic coat, was slightly enlarged, bulbous, and yielded on pressure a minute quantity of encephaloid matter. In conclusion, it should be stated that the lacrymal gland was preternaturally small, white, and indurated.

A considerable degree of nervous irritability, with vomiting, followed the operation, but was succeded by very little fever. The tumefaction of the eyelids was not greater than might have been expected. In a few days, a discharge of healthy pus came on, and in the course of two or three weeks the lids had contracted, granulations appeared, and the aspect of the parts was in all respects auspicious. She continued liable, however, to occasional attacks of chilliness, nausea, epigastric sinking, vomiting and severe pain in the head and socket. For sometime after the operation the integuments of the right side of the forehead were considerably reduced in their sensibility. In the month of January she left the Infirmary, and at this time, March, her situation is not known.

In his remarks on this case to the Clinical class, Prof. D. justified the operation on the ground, that, although a reproduction of encephaloid structure generally, follows the operation, such is not the invariable result; and that the removal of the morbid mass might prolong the existence of the patient, by preserving her from its reactive influence on the constitution. Moreover, the disease in this instance appeared to be the offspring of a local injury, the tumor seemed to be without attachments to the periosteum, and the muscles were evidently not disorganized as they were obedient to the will of the patient.

The Professor remarked, that Fungus Hæmatodes is an exceedingly common disease in the Western states, more common, indeed, than carcinoma, (if we except that of the female breast,) and that it is by no means confined to children and young persons, as some European writers intimate, for that he has known many cases of it in persons upwards of 40 years of age; and lately saw Prof. Parker extirpate a large encephaloid tumor from the side of a man in his 56th year.

The Professor observed, that although this malady is generally regarded as of constitutional origin, he had seen several cases in which it was preceded by mechanical violence, to the part; but in these instances there might have been a constitutional predisposition.

In a subsequent number we shall make known the termination of this case. We have lately learned that Bromly, whose arm was amputated in the Cincinnati Hospital, by Prof. McDowell, on the same day in which Mrs. J.'s eye was extirpated, and whose case was reported in the last number of the Journal, has got well. It will be recollected that his disease was Fungus Hæmatodes o the elbow joint.

Ulceration of the Duodenum. Formation of a Hepatico-Diaphragmatic Pouch.

J. R. aged 68 years, had been throughout life subject to rheumatism, but was otherwise of a robust constitution till within a few years of his death. He then became infirm, but not confined; lost a portion of flesh, acquired a dark sallow complexion, a bloodless skin, with defective perspiration, torpid bowels, and a weak, intermittent pulse. In the month of April, 1837, he was suddenly seized, in the evening, with an excruciating pain *in epigastrio*—such as is usually denominated cramp in the stomach, attended with reduction of temperature, and diminution in the ordinary force and fulness of his pulse. Large doses of stimulant and narcotic medicines, with external epigastric irritation, afforded some relief, and produced a certain degree of reaction, with a fit of vomiting. He was bled, but his pulse sunk under the operation; his bowels were opened with cathartics. Much debility ensued, with occasional flushes of fever, cough and dyspnœa. After a few days, his parotid glands became swollen and indurated. At length a hiccup supervened, and continued to recur till his death, which happened on the 13th of May, nineteen days after the attack of epigastric pain and spasm.

*Post-mortem Examination,* 30 *hours after death.*

This was made by Prof. Gross, in the presence of the attending physicians, Professors Drake and Rives, and the following appearances were observed.

Complexion dark and muddy; body considerably emaciated; costal cartilages ossified; left lung much smaller than natural, and universally adherent, by old cellular bands, to the ribs, but otherwise sound. Inferior lobe of the right lung united to the diaphragm and ribs, and covered with small masses and globules of lymph, so soft as to be easily wiped off with the finger. Its parenchymatous texture was extremely engorged, of a deep flesh color, infiltrated with globules of pus, and emitting, on being cut, a large quantity of thin, reddish, frothy serocity. A considerable portion was hepatized; and white creamy pus exuded in small quantity from the bronchial tubes. About four ounces of straw-colored serum were contained in the right pleuritic sac. The other two lobes were sound, and of a light greyish tint. The whole of the right lung, it should have been observed, was pushed up as high as the fourth rib by the liver. Cartilaginous rings of the trachea and bronchia ossified and very brittle; mucous membrane of the right tubes of a fleshy red color, extremely injected, with here and there a small ecchymosis, and filled with red frothy mucous.

*Heart*—Of the natural size and color, flabby and collapsed; coronary artery ossified for about three inches; walls of the left ventricle so soft as to break under the slightest effort of the finger; interventricular septum hypertrophous; fleshy columns sound; mitral valves covered with small specks of bone; semi-lunar valves of the aorta opake, wrinkled, and somewhat indurated; no remains of thymus gland.

Abdomen.—*Liver* of the natural size; about three-fourths of its anterior surface strongly adherent to the diaphragm; the adhesion extending about one inch beyond the longitudinal fissure, leaving the rest of the left lobe perfectly free and sound. On cutting through the diaphragm, the upper surface of which was of a dark color, and deeply injected, a flat circular pouch was discovered, a little more than six inches in diameter, formed by the organ just mentioned, and by the convex surface of the liver. This sac was capable of holding about twelve ounces of fluid, but was perfectly empty at the time of the examination. It was lined throughout, however, by a thick layer of lymph, of a yellow greenish color, from the intermixture, probably, of fœcal matter, which must have passed into it at different times from the duodenum, through an opening to which I shall presently more particularly allude. In cutting through this substance, where it covered the convex surface of the liver, the texture of the organ was found to be perfectly sound, excepting for about the fifth of an inch in depth, where it was somewhat softened, and of a dark bluish color. The outlet of this extraordinary pouch was formed by a small elliptical aperture, about five lines in diameter, situated at the anterior margin of the liver, nearly midway between the outer border of and the longitudinal fissure. This, after a tortuous course of about one inch and a half, communicated with the duodenum, by a circular opening six lines in diameter, with thick and well defined margins. The outlet here adverted to was seated one inch below the pylorus, on the upper wall of the duodenum, which, as well as the arch of the colon, adhered for several inches to the anterior edge of the liver, the coats of both tubes being perfectly healthy.

*Gall-bladder* concealed by dense cellular tissue, about the size of a large hen's egg, and filled with thick, yellow, greenish-bile; ducts pervious. Œsophagus, stomach, small and large intestines, sound, excepting the descending portion of the colon; the mucous membrane of which was injected, and of a dark reddish hue; the result probably of chronic irritation.

*Spleen* very small, of a dark mottled hue, and of good consistence, with numerous globules of lymph on its outer surface, and a small plate of bone, evidently seated in the fibrous tunic, one inch in length by one third in width, and of a pale yellowish color; internal structure sound.

Pancreas, kidneys, and urinary bladder healthy.

Parotid glands both very considerably enlarged, dense, of a greyish, gristly appearance, and pervaded with purulent matter, collected for the most part into little abscesses, the largest of which scarcely equalled the volume of a hazel nut. The pus was white, thick, and tenacious, like that of a scrofulous lymphatic gland. The weight of the right parotid was one ounce and two drachms; of the left one ounce and one drachm. The substance of the glands was scarcely any injected, a small vessel being only here and there perceptible. The swelling came on ten days before death; and, what is remarkable, the patient never complained of the slightest pain.

The interest of this case turns upon the successful efforts of nature to form a cyst or pouch between the liver and diaphragm, for the purpose of restraining the spread through the abdomen of the contents of the duodenum. A question presented is, whether this pouch was formed before or after the commencement of the epigastric pain? The perforation of the bowel might have then occurred; or it might bave happened long before, and the violent spasmodic pain which ushered in the attack might have been the consequence of the passage into it from the bowel, of something unusually irritating.

The morbid state of the right lung would seem to have been clearly referable, to the contiguity of that part to the inflamed diaphragm; while to the affection of the latter organ, the hiccup may be fairly ascribed. The suppuration of the parotid glands was, probably, an anomalous effect of calomel taken during his illness.

Fatal Apoplexy without engorgement or extravasation.

C. C., aged 44, an outstanding patient of the Cincinnati Hospital, arrived in the city, in a state of very reduced and deranged health, from bilious fever in the summer of 1837, on the shores of the Gulf of Mexico. When Prof. Drake was called to see him, February 4th, 1838, no intelligible account of his past situation could be obtained, as his mind was enfeebled and his association of ideas considerably impaired. His pulse was upwards of 100, and his complexion and general aspect indicated a constitution broken down by the fever of the preceding summer, relapsing, occasionally, upon him during the autumn and winter. His bowels were said by himself not to be costive, but this proved to be incorrect. His abdomen was distended. A prescription was made, and he was left for the night. Before entering, however, on the use of any thing, he walked from his room in the tavern, through a hall, to the supper table, at which he seated himself, but, before beginning to eat, fell senseless on the floor, and was carried to his bed. Prof. D. being summoned, found him in a state of deep insensibility, with stertorous respiration, and a pulse more than 120 in a minute. Venesection by a large orifice was immediately employed, and he lost nearly six pounds of blood, before his pulse began to flag. Large doses of calomel were then washed down his throat; enemata administered, and external irritants applied. He was bled again, purged very copiously, blistered, and, finally, under an apprehension, that the disease might be of miasmatic origin, according to the views of Dr. McCullough, the sulphate of quina was given in liberal doses. In about 36 hours his sensibilities were, to a certain degree, restored, and he began to speak. His left side had more power and mobility than the right. At length his mind became greatly excited—he saw external dangers—trembled with fear—and was occasionally excited with anger. Finally, convulsions came on, and after they had been many times repeated, he fell into a state of insensibility, and expired on the 4th day from the attack.

On the afternoon of the same day, the body was examined by Prof. Gross, who found the abdominal viscera sound, except a little engorgement of the spleen; the brain and its membranes unengorged, and without extravasation; but there were about two ounces of serum in the ventricles and at the base of the organ. This had probably been effused during the attack.

